# Gender differences in PTSD severity and pain outcomes: Baseline results from the LAMP trial

**DOI:** 10.1371/journal.pone.0293437

**Published:** 2024-05-16

**Authors:** Jessica K. Friedman, Brent C. Taylor, Emily Hagel Campbell, Kelli Allen, Ann Bangerter, Mariah Branson, Gert Bronfort, Collin Calvert, Lee J. S. Cross, Mary A. Driscoll, Ronni Evans, John E. Ferguson, Alex Haley, Sierra Hennessy, Laura A. Meis, Diana J. Burgess

**Affiliations:** 1 VA HSR&D Center for the Study of Healthcare Innovation, Implementation and Policy, VA Greater Los Angeles Health Care System (VAGLACS), Los Angeles, California, United States of America; 2 Center for Care Delivery and Outcomes Research, Minneapolis Veterans Affairs Healthcare System (MVAHCS), Minneapolis, Minnesota, United States of America; 3 University of Minnesota Medical School, Minneapolis, Minnesota, United States of America; 4 Center of Innovation to Accelerate Discovery and Practice Transformation, Durham VAHCS, Durham, North Carolina, United States of America; 5 Thurston Arthritis Research Center, University of North Carolina at Chapel Hill, Chapel Hill, North Carolina, United States of America; 6 Integrative Health & Wellbeing Research Program, Earl E. Bakken Center for Spirituality & Healing, School of Nursing, University of Minnesota, Minneapolis, Minnesota, United States of America; 7 VA Connecticut Healthcare System, West Haven, Connecticut, United States of America; 8 Yale School of Medicine, New Haven, Connecticut, United States of America; 9 Women’s Health Sciences Division, National Center for PTSD, Boston, Massachusetts, United States of America; University of São Paulo, BRAZIL

## Abstract

**Background:**

Post-traumatic stress disorder (PTSD) and chronic pain are highly prevalent comorbid conditions. Veterans dually burdened by PTSD and chronic pain experience more severe outcomes compared to either disorder alone. Few studies have enrolled enough women Veterans to test gender differences in pain outcomes [catastrophizing, intensity, interference] by the severity of PTSD symptoms.

**Aim:**

Examine gender differences in the association between PTSD symptoms and pain outcomes among Veterans enrolled in a chronic pain clinical trial.

**Methods:**

Participants were 421 men and 386 women Veterans with chronic pain who provided complete data on PTSD symptoms and pain outcomes. We used hierarchical linear regression models to examine gender differences in pain outcomes by PTSD symptoms.

**Results:**

Adjusted multivariable models indicated that PTSD symptoms were associated with higher levels of pain catastrophizing (0.57, 95% CI [0.51, 0.63]), pain intensity (0.30, 95% CI [0.24, 0.37]), and pain interference (0.46, 95% CI [0.39, 0.52]). No evidence suggesting gender differences in this association were found in either the crude or adjusted models (all interaction p-values<0.05).

**Conclusion:**

These findings may reflect the underlying mutual maintenance of these conditions whereby the sensation of pain could trigger PTSD symptoms, particularly if the trauma and pain are associated with the same event. Clinical implications and opportunities testing relevant treatments that may benefit both chronic pain and PTSD are discussed.

## Background

Post-traumatic stress disorder (PTSD) and chronic pain are highly prevalent comorbid conditions that affect the health and well-being of U.S Veterans [1, 2]. Those dually burdened by PTSD and chronic pain may experience more severe psychiatric functional impairments and greater health care utilization than those with either disorder alone [3–5]. Women Veterans, the fastest growing demographic of Veterans, experience disproportionally high rates of PTSD, 13%, compared to 6% of men Veterans, and between 11–78% experience chronic pain, compared to 9–50% of men Veterans [6–10]. Despite the growing body of knowledge on comorbid PTSD and chronic pain, few studies have enrolled enough women Veterans to test gender differences in pain outcomes (pain catastrophizing, interference, and intensity) in relation to PTSD symptoms. Understanding the potential gender variation in the severity of chronic pain outcomes associated with PTSD symptoms has implications for developing gender-sensitive assessment tools and treatment modalities for Veterans with PTSD and chronic pain.

Chronic pain is defined as pain lasting longer than three consecutive months and is associated with decreased quality of life, increased medical costs, and increased substance use [11–14]. In addition to the higher musculoskeletal pain reported by women Veterans, they are more likely to report moderate or severe chronic pain [15, 16]. At least one study found that women Veterans experienced greater pain intensity, decreased physical functioning, and more affective distress despite reporting better self-perceived health than men [17]. Additionally, gender role expectations and societal norms may influence how individuals perceive and communicate their pain experiences, potentially contributing to reported disparities in clinical pain assessment, diagnosis, and offered treatment modalities [18–20]. Finally, previous studies have revealed higher pain catastrophizing, interference and intensity levels in Veteran and civilian women compared to Veteran and civilian men [21, 22].

PTSD is a mental health disorder that may develop after individuals experience or witness a traumatic event and then subsequently experience a pattern of symptoms, including intrusion, avoidance, negative cognitions, and mood, or alterations in arousal and reactivity [23]. It is associated with an increased risk of additional mental health diagnoses, although most people who experience traumatic events do not ultimately develop PTSD. Men Veterans report proportionally more combat-related exposures linked to PTSD symptoms [24]. However, women Veterans who served in post-9/11 combat zones, such as Iraq and Afghanistan, report similar levels of perceived threat in the war zone as men [25, 26]. Approximately one in ten women Veterans overall, and one in four women Veterans who served during wartime, report being affected by PTSD [8].Women Veterans report higher incidence of exposure to traumatic events in both civilian life (e.g., adverse childhood experiences, sexual and/or physical assault, intimate partner violence) and miliary exposures (e.g., military sexual trauma, combat-related exposures) compared to both civilian women and men Veterans [27, 28]. The potential for cumulative impacts of traumatic exposures is associated with higher rates of PTSD and more severe PTSD symptoms in women Veterans compared to civilian women and men Veterans [27, 29–31].

Comorbid chronic pain and PTSD frequently co-occur in civilian and Veteran populations and are thought to have a bidirectional and mutually reinforcing relationship [32, 33]. Chronic pain and PTSD are thought to share common underlying mechanisms. The shared vulnerability and mutual maintenance theories have been used to explain the co-morbidity of these conditions. The shared vulnerability theory posits that people with comorbid PTSD and chronic pain share common factors, such as anxiety sensitivity, attentional bias, autonomic arousal, and psychobiological dysregulation, predisposing them to one or both conditions [32]. The mutual maintenance model posits that in addition to shared vulnerability factors (e.g., attentional biases, anxiety sensitivity), PTSD and chronic pain may exacerbate and reinforce each other [33]. This bidirectional influence may result in the experience of pain-related exacerbation of PTSD symptoms by retriggering recollections of painful injuries sustained during the traumatic event. Conversely, elevated PTSD symptoms may activate the body’s stress response and the experience of increased pain. The co-morbidity of chronic pain and PTSD has been found in treatment-seeking samples of PTSD and chronic pain patients [1, 32]. This mechanism has been well studied in predominantly male Veteran [34] and civilian [35] samples, which may obscure important differences in the manifestation and severity. While the findings of studies using Veteran samples support the central tenant of the mutual maintenance model, namely the mutually reinforcing and exacerbating influence of PTSD on chronic pain outcomes, very few have had adequate numbers of men and women Veterans to identify differential effects by gender [36]. The current study assesses whether gender differences exist in association between PTSD symptoms and pain outcomes in a comparative sample of men and women Veterans.

This manuscript reports the associations between probable PTSD symptoms and three dimensions of chronic pain outcomes: pain catastrophizing (negative thinking about one’s pain), pain intensity (how strong the pain feels), and pain interference (how one experiences pain, pain interference with daily activities), in a sample of U.S. Veterans diagnosed with a chronic pain condition. Specifically, we assessed gender differences in the association between probable PTSD and chronic pain outcomes. Based on known gendered differences in the nature of traumatic exposures in men and women Veterans, higher prevalence of PTSD symptoms, and evidence suggesting higher pain intensity and interference among women Veterans, the study team hypothesized that gender differences would be present, and women Veterans would present stronger associations between PTSD symptoms and chronic pain outcomes (pain intensity, interference, and catastrophizing). This study extends previous research on the associations between PTSD and chronic pain due to the unique gender composition of our data, which includes approximately equal numbers of men and women Veterans with chronic pain.

## Methods

### Study design and sample

This is a secondary analysis of baseline data from Veterans randomized for participation in the Learning to Apply Mindfulness to Pain (LAMP) study (*n* = 811). The LAMP study was a three-arm, multi-site, randomized pragmatic trial designed to assess the efficacy of multimodal delivery of two mindfulness-based interventions compared to usual care for Veterans with chronic pain at one of the three participating sites. Participants were recruited from the Minneapolis, Durham, and Greater Los Angeles Veterans Affairs Healthcare Systems. To be eligible, participants needed to have: 1) at least two qualifying pain diagnoses within the same pain category on at least two separate occasions at least 90 days apart during the previous two years documented in their electronic health record (EHR), 2) a pain duration of at least six months, a pain severity score of at least four during the previous week on a Likert scale of 0–10, and 3) access to a smartphone and the internet. Participants were excluded if they had: 1) new diagnoses of schizophrenia, bipolar disorder, major depressive disorder, current psychotic symptoms, suicidality, or other psychosis within the past 18 months (assessed by chart review); or 2) were currently enrolled in another pain study or a Mindfulness-Based Stress Reduction (MBSR) program (assessed by survey). Qualifying pain diagnoses were defined using International Classification of Diseases, Tenth Revision, Clinical Modification diagnostic codes (ICD-10-CM) and included: abdominal and bowel pain; back pain; bone infections; fibromyalgia and wide-spread muscle pain; fractures, contusions, sprains and strains; headache; infectious arthritic diseases; limb extremity pain, joint pain and arthritic disorders; musculoskeletal chest pain; neck pain; neuropathy; orofacial, ear, and temporomandibular disorder pain; other painful conditions; systemic disorders or diseases causing pain; urogenital, pelvic, and menstrual pain. A waiver of documentation of informed consent and the data collection reported herein was approved by the VA Central Institutional Review Board (IRB) (project 18–21). Participants were provided an information sheet about the study which included required consent form language and how to contact study staff with any questions. Additional study details are described in the protocol paper [37].

Of the participants (*n* = 1945) who met eligibility criteria, 1,737 completed the baseline survey, and 811 were randomized to one of the intervention arms. Due to an a priori study aim to examine gender differences, women Veterans were oversampled and comprised 48% of participants.

### Measures

#### Predictors

*PTSD symptoms* were assessed using the PTSD Checklist for *Diagnostic and Statistical Manual of Mental Disorders-Fifth Edition* (PCL-5) [38]. The PCL-5 is a 20-item self-report measure that assesses the presence and severity of PTSD symptoms over the past month (range, 0–80). Respondents are asked to rate how bothered they have been by each of the 20 items on a five-point Likert scale. Response options for all items are: “Not at all” (0), “A little bit” (1), “Moderately” (2), “Quite a bit” (3), or “Extremely” (4). Items are summed with scores of 31 or higher, indicating probable PTSD and a need for additional clinical assessment or PTSD treatment [39]. PCL-5 scores indicated excellent reliability (Cronbach’s α = 0.96), were standardized, and parameterized as continuous in the analysis.

*Outcomes*. *Pain Catastrophizing* was assessed using the Pain Catastrophizing Scale (PCS) [40]. The PCS is a 13-item self-report instrument that asks respondents to reflect on past painful experiences and indicate their thoughts and feelings in response to pain (range, 0–52). Response options for all items are: “Not at all” (0), “To a slight degree” (1), “To a moderate degree” (2), “To a great degree” (3), or “All the time” (4). Items are summed with scores of 30 or higher, representing a clinically significant level of pain catastrophizing [40]. PCS scores indicated excellent reliability (Cronbach’s α = 0.93), were standardized, and parameterized as continuous in analysis.

*Pain intensity and interference*. The Brief Pain Inventory (BPI) subscales were used to assess participant experiences of pain intensity (range, 0–10) and interference (range, 0–10) during the past week [41, 42]. Pain interference was assessed by seven items asking the extent to which pain interferes with general activity, mood, walking ability, normal work, relations with other persons, sleep, and enjoyment of life on an 11-point numeric rating scale from “Does not interfere” (0) to “Completely interferes” (10). Pain intensity was assessed by four items asking participants to rate their worst, least, average, and current pain severity for the past 1 month on an 11-point numeric rating scale from “No pain” (0) to “Pain as bad as you can imagine” (10). Each BPI subscale score indicated very good reliability (Cronbach’s α = 0.88), were standardized, and parameterized as continuous in analysis [43].

#### Covariates

*Gender* was defined based on the self-reported measure of gender on the study survey, with four response options (man, woman, another gender, decline to answer). Information recorded in the legacy EHR “birth-sex” variable, which can reflect either sex assigned at birth or self-reported gender identity and was used to recode the dichotomized gender variable for analysis as “woman” or “man” due to the small number of responses (five total) in other categories.

*Sociodemographic* variables included self-identified racial identity (“White,” “African American or Black,” “American Indian/Alaskan Native,” “Asian,” Native Hawaiian/Pacific Islander,” and “Multiracial”); ethnicity (“Hispanic or Latino” and “Not Hispanic or Latino”), highest level of educational attainment (“High school or less,” “Some college,” “Bachelor’s degree,” and “Master’s degree or beyond”), and age. The patient facility where participants were recruited (Minneapolis, Durham, or Greater Los Angeles VA Medical Centers) was also included in adjusted models.

### Analysis

First, we conducted descriptive analyses summarizing distributions of all variables overall and stratified by gender identity (Table 1). Crude and sociodemographic adjusted linear regression models were used to quantify the association between PTSD symptoms and each pain outcome (pain catastrophizing, intensity, and interference). We fit two sets of models (crude and adjusted) models to assess the association between PTSD symptoms and each pain outcome. We additionally assessed effect measure modification by gender (whether the association between PTSD symptoms and each pain outcome differed by gender identity) by adding an interaction term to each model. Two supplemental analyses were performed and are reported in S1 and S2 Tables in S1 File. The first added use of opioid medication for pain management in the past three months as a covariate (S1 Table in S1 File) and the second tested the effect of PTSD symptom clusters (i.e., re-experiencing, avoidance, negative alterations in cognitions and mood, hyper-arousal) on pain outcomes (S2 Table in S1 File). Results are reported in supplemental tables as they did not substantively change the point estimates, improve confidence interval precision, or provide meaningful new information on associations between PTSD symptoms and pain outcomes in our sample. Due to differences in the scale range of all outcomes, all estimates were standardized to represent the number of standard deviations of change in each pain outcome associated with a one standard deviation change in the exposure variable, PTSD symptoms. Statistical significance was assessed at alpha level p<0.05. All statistical analyses were conducted using Stata 18 (College Station, TX).

**Table 1:** Demographic characteristics.

	Full sample (N = 807)	Men (N = 421)	Women (N = 386)	p-value*
Age (mean, sd)	55 (12.9)	58 (12.6)	50 (11.8)	p<0.001
*Racial Identity*				p<0.001
African American/ Black	207 (26%)	70 (17%)	137 (36%)	
American Indian/ Alaskan Native	9 (1%)	5 (1%)	4 (1%)	
Asian	6 (1%)	3 (0.75%)	3 (0.75%)	
Multiracial	35 (4%)	15 (4%)	20 (5%)	
Native Hawaiian/ Pacific Islander	1 (0.1%)	*	1 (0.25%)	
White	545 (68%)	326 (78%)	219 (57%)	
*Ethnicity*				p = 0.102
Hispanic or Latino	51 (6%)	21 (5%)	30 (8%)	
Not Hispanic or Latino	755 (94%)	400 (95%)	355 (92%)	
*Employment Status*				p<0.001
Working now	333 (41%)	152 (36%)	181 (47%)	
Disabled	174 (22%)	86 (20%)	88 (23%)	
Retired	203 (25%)	142 (34%)	61 (16%)	
Other	97 (12%)	41 (10%)	56 15%)	
*Income*				p = 0.098
Live comfortably	247 (31%)	133 (32%)	114 (30%)	
Meet your basic expenses with a little left over for extras	343 (43%)	189 (45%)	154 (40%)	
Just meet your basic expenses	187 (23%)	88 (21%)	99 (26%)	
Don’t even have enough to meet basic expenses	30 (4%)	11 (3%)	19 (5%)	
*Education*				p = 0.007
High school or less	53 (7%)	36 (9%)	17 (4%)	
Some college	353 (44%)	196 (47%)	157 (41%)	
Bachelor’s degree	220 (27%)	109 (26%)	111 (29%)	
Master’s degree or more advance education	181 (22%)	80 (19%)	101 (26%)	
**Behavioral variables (Mean, SD)**				
Brief Pain Inventory (BPI)- Interference (0–10)	5.6 (2.0)	5.4 (1.9)	5.8 (2.0)	p = 0.002
Brief Pain Inventory (BPI)- Intensity (0–10)	5.5 (1.5)	5.4 (1.5)	5.7 (1.6)	p = 0.001
Pain catastrophizing (PCS) (0–52)	22.4 (11.3)	21.3 (11.4)	23.6 (11.1)	p = 0.004
PTSD (PCL-5) (0–80)	27.3 (19.5)	26.0 (19.3)	28.7 (19.6)	p = 0.046

*ttest or chisq test; (scale range)

### Results

A total of 807 participants provided complete data on PTSD, pain catastrophizing, pain intensity, and pain interference on the baseline survey (*n =* 807/811). Women in our sample were more likely to be employed, have four-year or more advanced degrees, be a member of a racially minoritized group, and be younger compared to men. Mean and standard deviations of scores for all outcome variables are also shown in Table 1. In bivariate analyses, women reported statistically significant higher mean scores for all pain outcomes and PTSD symptoms compared to men.

#### PTSD symptoms and pain outcomes

Table 2 shows the standardized estimates, 95% confidence intervals (CI), and results from the regression models examining the association between PTSD symptoms and pain outcomes (catastrophizing, intensity, and interference). Results are shown for both the crude and the model adjusted for sociodemographic characteristics. Results from the adjusted models indicated that PTSD symptoms were associated with higher levels of pain catastrophizing (0.57, 95% CI [0.51, 0.63]), pain intensity (0.30, 95% CI [0.24, 0.37]), and pain interference (0.46, 95% CI [0.39, 0.52]). No evidence suggesting gender differences in this association were found in either the crude or adjusted models (all interaction p-values<0.05).

**Table 2:** Associations between PTSD symptoms and pain outcomes in veterans.

	Pain Catastrophizing			Pain Intensity			Pain Interference		
	β	95% CI	Gender Interaction p-value	β	95% CI	Gender Interaction p-value	β	95% CI	Gender Interaction p-value
Model 1	0.60*	(0.55, 0.66)	0.233	0.33*	(0.26, 0.39)	0.670	0.47*	(0.41, 0.53)	0.594
Model 2	0.57*	(0.51, 0.63)	0.224	0.30*	(0.24, 0.37)	0.579	0.46*	(0.39, 0.52)	0.679

*p<0.001

## Discussion

This study investigated whether the associations between PTSD symptoms and pain catastrophizing, intensity, and interference differed in an approximately equal sample of women and men Veterans. While our bivariate results indicated higher average PTSD symptom scores, pain catastrophizing, pain intensity, and pain interference among women Veterans, gender differences were not significant in interaction models. Our results revealed that higher PTSD symptoms had the strongest effect on elevated pain catastrophizing symptoms, followed by pain interference and intensity in both men and women Veterans.

Co-morbidity between PTSD and chronic pain has been well-documented in civilian [44] and Veteran samples [4, 45]. Previous studies assessing the association between PTSD symptoms and pain outcomes in cut-points among Veteran (predominantly men >80%) samples have observed overall higher levels of pain catastrophizing, interference, and intensity [4, 5, 35, 46]. The inequity in the gender distribution in these studies has left unanswered questions regarding gender differences, given gendered variability in the timing and type of trauma exposure(s) that resulted in PTSD symptoms [26, 47]. While our results support previous findings, showing robust statistical associations between PTSD symptoms and pain catastrophizing, interference, and intensity, we did not find evidence of gender differences in these associations in our gender balanced sample [4, 5, 45, 48].

The present study provides a unique contribution to the existing literature due to the size and approximately equal proportions of men and women Veterans that allowed tests of heterogeneity of effects in this association by gender. The ability to investigate gender differences regarding pain and PTSD in previous Veteran samples has been hampered by poor recruitment and retention of women in VA clinical trials, which typically average only 3–22% women [36, 49]. The lack of gender differences, may reflect the underlying mechanism of the mutual maintenance model whereby the sensation of pain could trigger PTSD symptoms (intrusions, avoidance, arousal), particularly if the trauma and pain are associated with the same event and this mechanism operates similarly in men and women [32, 50]. Additionally, although statistically different, the baseline differences in PTSD symptom severity for men and women were small (> 5 points), suggesting this difference unlikely to be clinically meaningful. In the case of pain catastrophizing, this may reflect the tendency to magnify the threat value of pain, thereby increasing feelings of helplessness and difficulty inhibiting pain-related thoughts [51]. Alternatively, the lack of gender differences in these associations may be in part due to the restriction that all Veterans in the current analysis needed to meet inclusion criteria for entry into the LAMP clinical trial, which may make them more similar compared to the overall Veteran patient population. Eligibility criteria for this clinical trial screened out those with recent diagnosis of a mental disorder (e.g., major depressive disorder) or suicidality, which may have resulted in a clinical sample that is less psychiatrically severe compared to a typical sample of Veterans seeking chronic pain care. These restrictions may obscure gender differences present in more psychiatrically severe patients with comorbid chronic pain conditions.

Elevated levels of pain catastrophizing, intensity, and interference in people with probable PTSD, highlight the need for interdisciplinary care modalities to target both PTSD and chronic pain symptoms. There have been mixed results regarding the efficacy of cognitive-based therapies in reducing comorbid PTSD and pain, with interventions generally demonstrating greater reduction in PTSD symptoms with little change in pain interference and intensity [45, 52–54]. Mindfulness-based therapies have demonstrated reductions in both PTSD and chronic pain outcomes, indicating their promise as an effective treatment for people with comorbid PTSD and chronic pain [55–59]. Finally, Veterans with comorbid chronic pain and PTSD may specifically benefit from integrated and trauma-informed treatment modalities that recognize the mutually reinforcing relationship between PTSD and pain [60]. As the VA continues to develop and test multimodal care for Veterans with comorbid trauma and pain conditions, careful attention will be required to evaluate gender-sensitive treatment options (e.g. integrated into women’s clinic, online options) to ensure women Veterans feel comfortable accessing the required care to address these highly co-prevalent conditions [61, 62].

This study contributes to the literature by assessing associations between probable PTSD and pain outcomes as well as findings related to gender differences in a Veteran population with highly prevalent PTSD and comorbid chronic pain. Some limitations must be considered when interpreting our results. First, we were unable to account for gender differences in the onset of trauma, or exposure to specific traumatic events across the life-course (e.g., adverse childhood experience, sexual assault, service-related exposures), where the timing, frequency, and severity may relate to the onset of chronic pain symptoms. Second, we were unable to tease apart the proportion of Veterans with service-connected disability specifically tied to either chronic pain, PTSD symptoms, or both conditions compared to other types of service-related disabilities, as these Veterans may differ in their treatment-seeking behavior and willingness to enroll in a clinical trial. Third, while use of self-reported gender has a number of strengths, it limits our ability to generate hypotheses regarding the potential for biological sex differences (e.g., due to neuroendocrine system differences). Additional research is warranted to understand both the unique health needs and outcomes of Veterans who identify as non-binary and biological sex differences. Relatedly, five Veterans had either missing self-reported gender (three) or selected other genders (two). VA hospital record data was used for these individuals, which may or may not have been consistent with their self-identified gender. Finally, this analysis was conducted on a sample of participants who volunteered to participate in a mindfulness study for chronic pain. Therefore, findings may not generalize to the broader population of Veterans with chronic pain. Treatment-seeking Veterans with chronic pain may be different in important ways from their non-treatment seeking counterparts. Likewise, Veterans opting into a clinical trial of a mindfulness-based treatment for chronic pain may be different from those who did not opt into our study. Taken together, these differences may have inadvertently introduced selection bias into our study. Despite these limitations, our study has several strengths, including completeness of data and the inclusion of survey and electronic health record data from a large, diverse, and balanced sample of Veterans with chronic pain. Finally, this uniquely large and gender-balanced study cohort of Veterans allowed for analyses into potential gender differences in pain outcomes by the presence of probable PTSD.

## Conclusion

This study found that PTSD symptoms were associated with elevated levels of pain catastrophizing, pain intensity, and pain interference in women and men Veterans. We did not find any statistically significant gender differences in the associations between PTSD symptoms and pain outcomes. The unique gender composition of this sample created an opportunity to test these differences, addressing a limitation present in other Veteran samples. Our results contribute to research in Veterans aimed at understanding shared underlying mechanisms of this co-morbidity and testing relevant treatments that may concurrently benefit both chronic pain and PTSD.

## Supporting information

S1 File Supplemental analyses can be referenced in S1 and S2 Tables in S1 File. (DOCX)
