# Supplementary material for: Gender differences in PTSD severity and pain outcomes: Baseline results from the LAMP trial
Source: PLoS One. 2024 May 16;19(5):e0293437. doi: 10.1371/journal.pone.0293437 (PMC11098421; doi:10.1371/journal.pone.0293437)
Supplement: S1 File — (DOCX) [file pone.0293437.s001.docx]

**Supplemental Table 1: Additional opioid medication covariate**

|  | **Pain Catastrophizing** | | | | **Pain Intensity** | | | | **Pain Interference** | | | |
| --- | --- | --- | --- | --- | --- | --- | --- | --- | --- | --- | --- | --- |
|  | β | 95% CI | p-value | Gender Interaction p-value | β | 95% CI | p-value | Gender Interaction p-value | β | 95% CI | p-value | Gender Interaction p-value |
| Model 1 | 0.60 | (0.55, 0.66) | <0.01 | 0.23 | 0.33 | (0.26, 0.39) | <0.01 | 0.67 | 0.47 | (0.41, 0.53) | <0.01 | 0.59 |
| Model 2 | 0.57 | (0.51, 0.63) | <0.01 | 0.22 | 0.30 | (0.24, 0.37) | <0.01 | 0.58 | 0.46 | (0.39, 0.52) | <0.01 | 0.68 |
| Model 3 | 0.50 | (0.46, 0.61) | <0.01 | 0.27 | 0.30 | (0.23, 0.41) | <0.01 | 0.51 | 0.40 | (0.36, 0.53) | <0.01 | 0.76 |
|  |  |  |  |  |  |  |  |  |  |  |  |  |
| Model 1 - crude - no adjustment | | | |  |  |  |  |  |  |  |  |  |
| Model 2- sociodemographic adjusted model | | | | |  |  |  |  |  |  |  |  |
| Model 3 with Opioid medication for pain management in past 3 months | | | | | | | |  |  |  |  |  |

| **Supplemental Table 2: Effect of PTSD clusters on pain outcomes** | | | | | | | | | |  |  |  |
| --- | --- | --- | --- | --- | --- | --- | --- | --- | --- | --- | --- | --- |
|  | **Pain Catastrophizing** | | | | **Pain Intensity** | | | | **Pain Interference** | | | |
| Predictors | β | 95% CI | p-value | Gender Interaction p-value | β | 95% CI | p-value | Gender Interaction p-value | β | 95% CI | p-value | Gender Interaction p-value |
| Re-experiencing | 0.49 | (0.41, 0.58) | <0.001 | 0.448 | 0.31 | (0.22, 0.40) | <0.001 | 0.538 | 0.41 | (0.32, 0.50) | <0.001 | 0.861 |
| Avoidance | 0.40 | (0.32, 0.49) | <0.001 | 0.585 | 0.27 | (0.18, 0.36) | <0.001 | 0.477 | 0.33 | (0.24, 0.42) | <0.001 | 0.792 |
| Negative alterations in cognition and mood | 0.50 | (0.42, 0.58) | <0.001 | 0.151 | 0.26 | (0.17, 0.35) | <0.001 | 0.634 | 0.41 | (0.32, 0.50) | <0.001 | 0.852 |
| Hyperarousal | 0.53 | (0.44, 0.61) | <0.001 | 0.791 | 0.34 | (0.24, 0.43) | <0.001 | 0.415 | 0.44 | (0.34, 0.53) | <0.001 | 0.877 |
| *Models adjusted for sociodemographic and study design characteristics | | | | | | | | | |  |  |  |
